# Gelsolin activity controls efficient early HIV-1 infection

**DOI:** 10.1186/1742-4690-10-39

**Published:** 2013-04-10

**Authors:** Laura García-Expósito, Serena Ziglio, Jonathan Barroso-González, Laura de Armas-Rillo, María-Soledad Valera, Donato Zipeto, José-David Machado, Agustín Valenzuela-Fernández

**Affiliations:** 1 Cellular and Viral Immunology Lab, Department of Physical Medicine and Pharmacology, School of Medicine, University of La Laguna (ULL), Campus de Ofra s/n, Tenerife, 38071, Spain; 2 Laboratory of Molecular Virology, Section of Biology and Genetics, Department of Life and Reproduction Sciences, School of Medicine, University of Verona, Strada le Grazie 8, Verona, 37134, Italy; 3 Pharmacology Unit, Department of Physical Medicine and Pharmacology, School of Medicine, University of La Laguna (ULL), Campus de Ofra s/n, Tenerife, 38071, Spain

**Keywords:** Gelsolin, Actin-severing activity, Perturbed-actin dynamics and receptors clustering, Inhibition of early HIV-1 infection

## Abstract

**Background:**

HIV-1 entry into target lymphocytes requires the activity of actin adaptors that stabilize and reorganize cortical F-actin, like moesin and filamin-A. These alterations are necessary for the redistribution of CD4-CXCR4/CCR5 to one pole of the cell, a process that increases the probability of HIV-1 Envelope (Env)-CD4/co-receptor interactions and that generates the tension at the plasma membrane necessary to potentiate fusion pore formation, thereby favouring early HIV-1 infection. However, it remains unclear whether the dynamic processing of F-actin and the amount of cortical actin available during the initial virus-cell contact are required to such events.

**Results:**

Here we show that gelsolin restructures cortical F-actin during HIV-1 Env-gp120-mediated signalling, without affecting cell-surface expression of receptors or viral co-receptor signalling. Remarkably, efficient HIV-1 Env-mediated membrane fusion and infection of permissive lymphocytes were impaired when gelsolin was either overexpressed or silenced, which led to a loss or gain of cortical actin, respectively. Indeed, HIV-1 Env-gp120-induced F-actin reorganization and viral receptor capping were impaired under these experimental conditions. Moreover, gelsolin knockdown promoted HIV-1 Env-gp120-mediated aberrant pseudopodia formation. These perturbed-actin events are responsible for the inhibition of early HIV-1 infection.

**Conclusions:**

For the first time we provide evidence that through its severing of cortical actin, and by controlling the amount of actin available for reorganization during HIV-1 Env-mediated viral fusion, entry and infection, gelsolin can constitute a barrier that restricts HIV-1 infection of CD4+ lymphocytes in a pre-fusion step. These findings provide important insights into the complex molecular and actin-associated dynamics events that underlie early viral infection. Thus, we propose that gelsolin is a new factor that can limit HIV-1 infection acting at a pre-fusion step, and accordingly, cell-signals that regulate gelsolin expression and/or its actin-severing activity may be crucial to combat HIV-1 infection.

## Background

The host cell cytoskeleton plays a key role in the life cycle of viral pathogens, the propagation of which depends on several mandatory intracellular stages. Accordingly, the human immunodeficiency virus type 1 (HIV-1) has evolved strategies to exploit and modulate the actin cytoskeleton for its own purposes [1-3]. The entry of HIV-1 into a target cell requires the interaction of multiple receptor and co-receptor molecules with each viral envelope (Env) trimer in order to promote the formation of fusion pores [4,5]. The HIV-1 Env gp120 protein regulates the initial attachment of viral particles to target cells through its association with CD4 and either CXCR4 or CCR5, which results in the exposure of the fusion-promoting peptide of the Env gp41 region. Moreover, HIV-1 induces the activation of moesin, an actin-adaptor protein, to promote the redistribution of F-actin and CD4/CXCR4 clustering on the cell surface at viral entry points [6]. These events are crucial to ensure efficient HIV-1/cell-membrane fusion, viral entry and infection in T lymphocytes [1,6]. Thus, there is a clear kinetic and spatial association between moesin phosphorylation and F-actin-mediated viral receptor capping that promotes HIV-1 infection.

HIV-1 Env-gp120-dependent actin remodelling also involves the actin-crosslinker filamin-A, Rho-A and Rac guanosine-triphosphatases (GTPases), and the actin-depolymerization factors cofilin [7-9] and syntenin-1[10]. Filamin-A binds to the cytoplasmic tails of CD4 and viral co-receptors, and the ensuing HIV-1 Env-gp120-dependent signalling activates Rho-A [7] and Rac1 [9]. Both Rho-A and the Rac GTPase activate the LIM domain kinase, which phosphorylates and inactivates cofilin, in turn triggering early actin polymerization [9]. Together, these processes increase the probability of HIV-1 Env-CD4/co-receptor interactions, potentiating fusion pore formation and hence, HIV-1 viral fusion and infection (reviewed in [1-3]). Cofilin and syntenin-1 appear to depolymerise F-actin in a post-fusion step, allowing the entry of the viral capsid into target cells [8,10]. However, the HIV-1 Env signalling that lies upstream of the Rho-A/Rac-LIMK-cofilin and syntenin-1 pathways remain poorly understood [10-13], and the identity of the kinase that phosphorylates moesin in the ERM complex is unknown [6]. Likewise, the processes underlying the recruitment and activation of the different actin remodelling factors due to HIV-1 Env signalling through CD4 and its co-receptors remain to be elucidated.

Gelsolin belongs to a superfamily of actin binding proteins expressed in all eukaryotes that includes villin, adseverin (also known as scinderin), CapG, flil and severin (also known as fragmin: reviewed in [14]). All members of this family contain at least one 120 amino acid structural repeat and many have three or six gelsolin repeats [15]. Gelsolin is an 80-kDa protein consisting of two tandem homologous fragments (segments 1–3 and 4–6) that each contains three repeats [16]. This protein trims large actin filaments, promoting actin cytoskeleton reorganization, and it alters plasma membrane morphology to generate motile filopodia and lamellipodia [17-19].

Given the actin-severing activity of gelsolin and the fundamental role of HIV-1 Env-gp120-orchestrated cortical actin reorganization in virus-mediated receptor aggregation, as well as in efficient HIV-1 fusion and entry, we investigated the functional role of intracellular gelsolin in early HIV-1 infection in permissive lymphocytes. We found that overexpression of functional intracellular gelsolin or specific silencing of endogenous gelsolin expression restricts HIV-1 entry and infection by altering the amounts of cortical F-actin, and by perturbing F-actin dynamics and the associated changes in pseudopodium morphology. We observed that these events negatively affect HIV-1 Env-mediated receptor clustering, membrane fusion and efficient viral entry and infection. Based on these findings, we propose that gelsolin can restrict early HIV-1 infection acting at a pre-fusion step, and altering the stability and dynamics of the cortical actin cytoskeleton in permissive lymphocytes.

## Results

### The actin-severing enzyme gelsolin is expressed in the cytoplasm and cortex of cells, where it regulates F-actin levels

**Figure 1:**
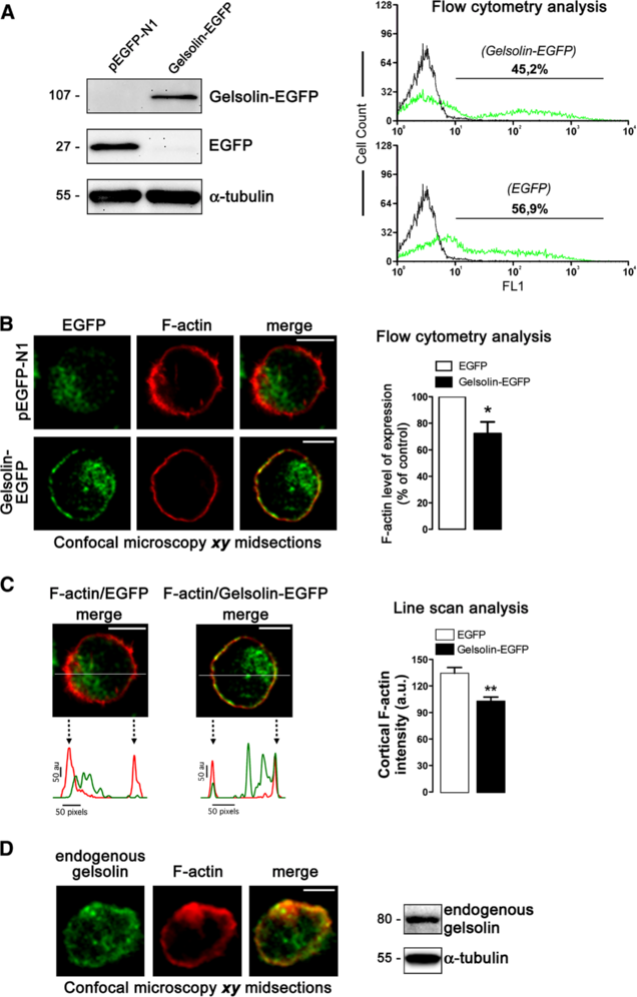
**Expression of functionally overexpressed or endogenous gelsolin and its effects on cortical F-actin.** (**A**) *Left*, Western blot of gelsolin-EGFP expression in CEM.NKR-CCR5 cells. Gelsolin-EGFP fusion protein was stable and did not undergo any processing of its C-terminal EGFP tag. α-tubulin and pEGFP-N1 are controls for total protein and free EGFP, respectively. *Right*, Quantification of gelsolin-EGFP (45.2%) and free EGFP (56.9%) expressing cells by flow cytometry. One representative experiment of three is shown. (**B**) *Left*, a series of confocal images, ***xy*** midsections, showing the distribution of overexpressed gelsolin-EGFP. F-actin, free EGFP and merged images for F-actin/gelsolin-EGFP co-localization at cell-surface are shown. One representative experiment of three different experiments is shown. *Right*, flow cytometry analysis of F-actin in EGFP- (control, taken as 100% F-actin expression) or gelsolin-EGFP- expressing cells. Data are mean ± s.e.m of three independent experiments: *p < 0.05, Student’s *t-*test. (**C**) *Left*, line scans show the fluorescence intensity profiles of cortical F-actin and the overexpressed free EGFP or gelsolin-EGFP proteins. Images correspond to the results presented in the merged images in panel **B**, analysed along the horizontal line drawn in panel **C**. *Right*, quantification of the fluorescence intensity profiles of cortical F-actin in a series of cells (12 cells per series) analysed by line scanning confocal microscopy (8 cortical points analyzed per cell). Data are mean ± s.e.m. of three independent experiments: **p < 0.01, *Student’s t-test*. (**D**) *Left*, a series of confocal images showing the distribution of endogenous gelsolin. F-actin and merged images for F-actin/gelsolin co-localization at cell-surface are shown. One representative experiment of three is shown. *Right,* Western blot analysis of endogenous gelsolin and F-actin expression. α-tubulin is a control of total protein expression. One representative experiment of three is shown. In **B**, **C** and **D**, scale bar = 5 μm.

To explore the roles of gelsolin and its F-actin severing activity in early HIV-1 entry and infection, we used a C-terminal enhanced green fluorescent protein (EGFP)-tagged wild type gelsolin (gelsolin-EGFP) derived from the permissive CEM.NKR-CCR5 T cell line. Transient expression of the gelsolin-EGFP construct (Figure 1A) in these permissive lymphocytes was confirmed in Western blots and by flow cytometry. Fluorescence confocal microscopy revealed that cortical gelsolin-EGFP co-localizes with cortical F-actin in the cytoplasm of CEM.NKR-CCR5 cells (Figure 1B, C), a similar distribution to that observed for endogenous gelsolin (Figure 1D). Transfection of a construct encoding the EGFP protein alone (as a control) revealed a clear cytoplasmic distribution, with no expression associated with the plasma membrane or actin filaments (Figure 1B, *pEGFP-N1 series of images*). In T cells transfected with gelsolin-EGFP, F-actin expression was diminished, as monitored by the intensity of the labelling of cortical F-actin, which was weaker than in cells expressing EGFP alone (Figure 1C, *line scan analysis and quantification histograms*) and in non-transfected cells (Figure 1D). These data were confirmed by quantifying total levels of F-actin expression by flow cytometry (Figure 1B, *right histogram*), which revealed that gelsolin-EGFP overexpression promoted actin filament severing and decreased the levels of cortical F-actin in CEM.NKR-CCR5 permissive lymphocytes. Our data demonstrate that the functional gelsolin construct and the endogenous gelsolin protein both co-localize with F-actin, in agreement with previous reports describing the binding of gelsolin to actin filaments, even during actin filament processing [20,21].

### Gelsolin restricts HIV-1 entry and infection in permissive lymphocytes, independently of viral tropism

**Figure 2:**
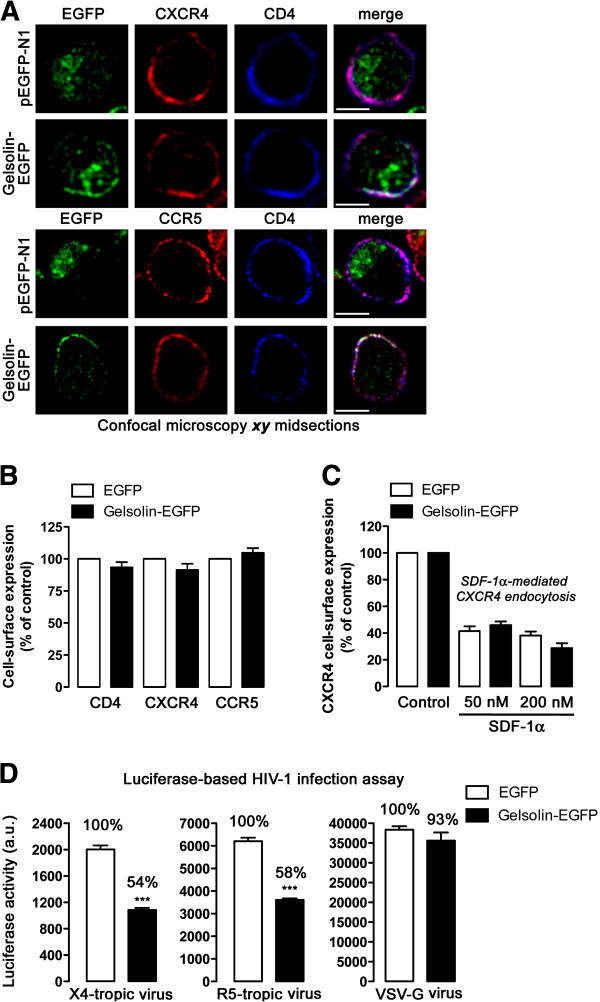
**Functional gelsolin overexpression impairs HIV-1 entry and infection in permissive lymphocytes, regardless of viral tropism.** (**A**) CD4 and CXCR4 (top images) or CCR5 (bottom images) distribution in uninfected CEM.NKR-CCR5 cells transfected with free EGFP (pEGFP-N1) or gelsolin-EGFP. Merged confocal microscopy images show the co-localization of CD4-CXCR4/gelsolin-EGFP or CD4-CCR5/gelsolin-EGFP at the plasma membrane. Scale bar = 5 μm; one representative experiment of three is shown, always analyzing 200 cells. (**B**) Flow cytometry analysis of the effect of gelsolin-EGFP or EGFP overexpression (control, 100% viral receptor expression in pEGFP-N1-transfected cells) on CD4, CXCR4 and CCR5 cell-surface expression. (**C**) Effect of gelsolin-EGFP overexpression on SDF-1α-induced CXCR4 endocytosis. Control (EGFP-transfected cells) and gelsolin-EGFP expressing cells were exposed to SDF-1α (50 nM and 200 nM), as described in Methods. CXCR4 expression was subsequently analyzed by flow cytometry in control/EGFP + and gelsolin-EGFP + cells using PE-conjugated specific mAbs against cell-surface CXCR4. The data are expressed as a percentage of CXCR4 expression in the absence of SDF-1α (control). (**D**) Luciferase assay of viral entry and infection by X4- and R5-tropic HIV-1 viral particles in gelsolin-EGFP transfected cells (EGFP-expressing cells were used as controls, and represent 100% viral entry and infection). All the data were corrected using the non-productive infection values (baseline) obtained in the presence of a neutralizing anti-CD4 mAb (5 μg/mL) under the same experimental conditions. A luciferase-based assay, with equal inputs of pseudotyped VSV-G virions was used to control the specificity of gelsolin-mediated effects on HIV-1 viral entry and infection. These data were corrected with non-productive baseline values obtained using non-infective virus-like particles lacking VSV-G-Env under the same experimental conditions. In **B**-**D** data are mean ± s.e.m. of three independent experiments carried out in triplicate: *** p < 0.001, Student’s *t-*test.

Since HIV-1 Env-gp120-induced reorganization of cortical actin has been proposed to be fundamental to promote efficient HIV-1 viral entry and infection [6-9], we therefore examined the effect of gelsolin overexpression on HIV-1 entry and infection. Overexpression of gelsolin-EGFP did not affect the cellular distribution or the cell-surface expression of CD4, CXCR4 or CCR5, the receptors required for HIV-1 infection (Figures 2A, B, respectively). Moreover, no alterations in ligand-induced internalization were observed in cells overexpressing gelsolin, indicating that these viral co-receptors were fully functional (Figure 2C).

We next studied early HIV-1 infection using single-cycle viruses bearing the *Luc*-reporter gene in permissive lymphocytes overexpressing functional gelsolin. These non-replicative viruses enable HIV-1 entry and infection to be monitored and quantified [6,22-24], excluding any potential influence of the late stages of the infection cycle. When X4- or R5-tropic HIV-1 viral particles were incubated with CEM.NKR-CCR5 permissive cells, HIV-1 entry and infection was significantly impaired in gelsolin-EGFP-expressing cells as compared with control cells (Figure 2D), with a similar impairment of both the X4- or R5-tropic HIV-1 viral strains (Figure 2D; *54% and 58% of X4-tropic and R5-tropic infection, respectively*). Thus, the actin-severing gelsolin protein modulated HIV-1 entry and infection regardless of viral tropism, indicating that the inhibitory effect of gelsolin on early HIV-1 infection is not related to defective signalling or decreased cell-surface expression of viral co-receptors.

We also assayed the effect of gelsolin overexpression on pseudotyped virions carrying the vesicular stomatitis virus (VSV)-G Env protein [6,22-24] that enter target cells using the clathrin-endocytic lower-pH pathway selected by VSV-G Env [24,25]. Entry and infection of these pseudotyped viral particles, using equal viral inputs for these virions and that for X4- and R5-tropic HIV-1 strains, were not significantly affected by gelsolin overexpression (Figure 2D).

Together, these observations suggest that overexpression of functional gelsolin restricts HIV-1 entry and infection of permissive lymphocytes regardless of viral tropism. It is conceivable that overexpression of gelsolin perturbs the HIV-1 Env-gp120-mediated cortical actin reorganization and dynamics, a process that may also be affected by a reduction in the pool of cortical actin. To address this issue, we studied the role of gelsolin in HIV-1 Env-mediated actin reorganization.

#### Gelsolin overexpression impairs HIV-1 Env-gp120-induced cortical F-actin reorganization and capping

**Figure 3:**
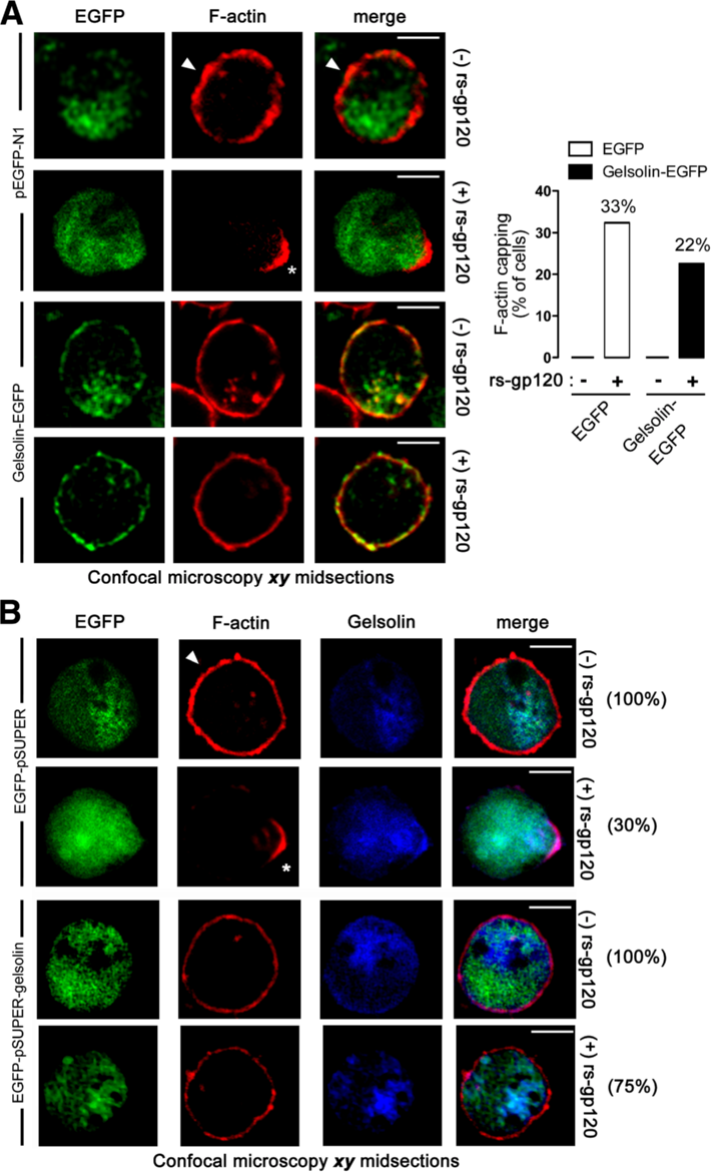
**Effect of gelsolin overexpression on HIV-1 Env-gp120-induced cortical F-actin redistribution in permissive lymphocytes.** (**A**) Confocal analysis of HIV-1 Env-gp120-induced F-actin redistribution in CEM.NKR-CCR5 cells expressing free EGFP (controls) or gelsolin-EGFP. In control cells, rs-gp120_IIIB_ (5 μg/mL) triggered F-actin capping (see white asterisk). F-actin expression was clearly visible in control conditions (see white arrowhead in pEGFP-N1 images) but was reduced in cells expressing gelsolin-EGFP. Merged images show the cortical co-localization of F-actin and gelsolin-EGFP or free EGFP. *Right histograms*, rs-gp120_IIIB_-triggered cortical F-actin capping is represented as the percentage of 200 cells counted for each experimental condition. Scale bar = 5 μm. A series of ***xy*** midsections from one representative experiment out of three is shown. (**B**) Similar data was obtained by confocal analysis of HIV-1 Env-gp120-induced F-actin redistribution in CEM.NKR-CCR5 cells expressing free EGFP (controls) or co-expressing both free gelsolin and EGFP. In control cells, rs-gp120_IIIB_ (5 μg/mL) triggered F-actin capping (see white asterisk). F-actin expression was clearly visible in control conditions (see white arrowhead in EGFP-pSUPER images) but was reduced in cells expressing EGFP-pSUPER-gelsolin. Merged images show the cortical co-localization of F-actin and gelsolin or free EGFP. Cortical F-actin capping (only observed in EGFP-pSUPER condition) or distribution is represented as the percentage of 200 cells counted for each experimental condition. Scale bar = 5 μm. A series of ***xy*** midsections from one representative experiment out of three is shown.

The role of gelsolin in HIV-1 Env-gp120-mediated F-actin reorganization was studied by transiently nucleofecting permissive lymphocytes with gelsolin-EGFP and then incubating them with functional, recombinant soluble HIV-1 Env-gp120 viral protein (rs-gp120_IIIB_). This viral protein promotes a reorganization of cortical actin to one pole of the cell in a CD4-dependent manner [6,22], as evident when cells overexpressing EGFP alone were exposed to the HIV-1 Env-gp120 protein (Figure 3A, pEGFP-N1 images; quantified in histograms, 33% of cells). However, the cortical actin clustering induced by rs-gp120_IIIB_ was impaired in permissive lymphocytes overexpressing gelsolin-EGFP (Figure 3A, gelsolin-EGFP series of images, quantified in histograms, 22% of cells). We next analyzed the HIV-1 Env-gp120-mediated F-actin reorganization in permissive lymphocytes transiently expressing both free EGFP and gesolin (encoded in the same EGFP-pSUPER-gelsolin plasmid) or free EGFP alone (encoded in a control EGFP-pSUPER plasmid), in order to explore whether or not the EGFP tag alters or conditions the observed effects attributed to gelsolin. HIV-1 Env-gp120-induced F-actin capping was similarly observed in control cells, expressing only free EGFP (Figure 3B, 30% of cells). As occurred in cells over-expressing gelsolin-EGFP (Figure 3A, 78% of non-reactive cells), the cortical actin clustering induced by rs-gp120_IIIB_ was impaired in permissive lymphocytes co-overexpressing free gelsolin and EGFP (Figure 3B, EGFP-pSUPER-gelsolin series of images; 75% of non reactive cells). Some endogenous gelsolin was detected within the F-actin capping region in this case (Figure 3B, see Gelsolin and F-actin in the EGFP-pSUPER (+) rs-gp120 series of images), but was not tightly restricted to the region of the actin cap, and endogenous gelsolin was also observed in the cytoplasm and at the plasma membrane of these activated cells. Therefore, over-expression of free gelsolin inhibits rs-gp120_IIIB_-induced F-actin reorganization and capping to a pole of the cell (Figure 3B). The EGFP moiety on gelsolin-EGFP does not appear to be responsible of or to significantly alter the effect exerted by over-expressed gelsolin-EGFP on HIV-1 Env-gp120-mediated F-actin reorganization and capping. Taken together these data suggest that efficient early HIV-1 infection requires the appropriate amount of cortical actin to be reorganized in a specific manner during the initial virus-cell contacts. Both these processes appear to be influenced by actin-severing gelsolin, suggesting that gelsolin activity can restrict HIV-1 entry and infection. Therefore, both overexpression of functional gelsolin or specific knockdown of endogenous gelsolin negatively affects HIV-1 Env-mediated actin dynamics, entry and infection at a pre-fusion step.

### Overexpression of functional gelsolin inhibits HIV-1 Env-gp120-mediated viral receptor redistribution and clustering

**Figure 4:**
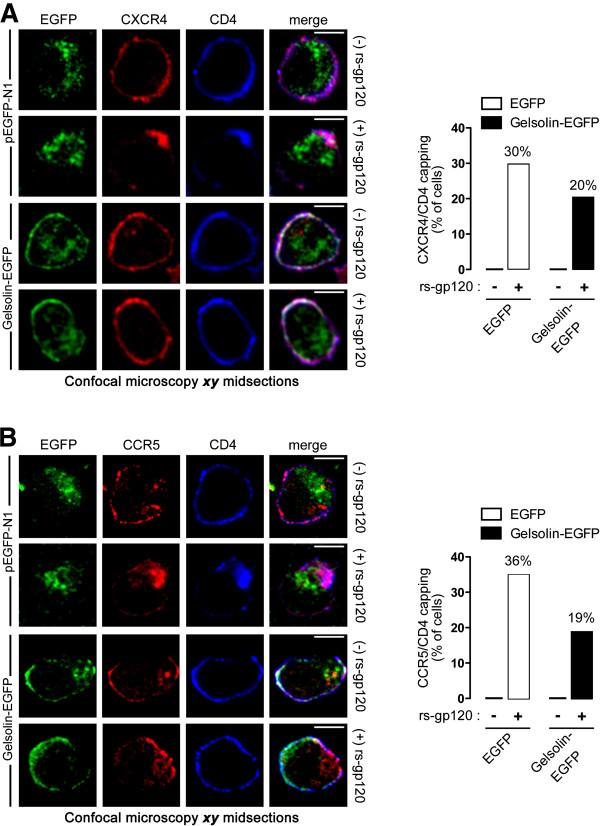
**Gelsolin functional overexpression impairs HIV-1 Env-gp120-mediated CD4-CXCR4 and CD4-CCR5 redistribution in permissive lymphocytes.** (**A**) *Left*, HIV-1 Env-gp120-induced redistribution of CD4-CXCR4 to the cell pole in CEM.NKR-CCR5 cells expressing gelsolin-EGFP or EGFP, 1 hour of after exposure to rs-gp120_IIIB_ (5 μg/mL) at 37°C. A series of ***xy*** midsections and merged images for CD4-CXCR4/gelsolin-EGFP co-localization at cell-surface are shown. Scale bar = 5 μm. One representative experiment of three is shown. *Right,* HIV-1 Env-gp120-induced CD4-CXCR4 capping is reflected as the percentage of 200 cells counted for each experimental condition, where the data represent the results of three independent experiments. (**B**) Analysis and quantification of HIV-1 Env-gp120-induced CD4-CCR5 redistribution in permissive cells expressing gelsolin-EGFP or EGFP. A series of ***xy*** midsections and merged images show the co-localization of CD4-CCR5/gelsolin-EGFP at the cell surface. Scale bar = 5 μm. One representative experiment of three is shown. *Right,* HIV-1 Env-gp120-induced CD4-CCR5 capping is shown as the percentage of 200 cells counted for each experimental condition, where the data represent the results of three independent experiments.

Confocal microscopy revealed significant CD4-CXCR4 clustering at one pole of the cell upon rs-gp120_IIIB_-mediated stimulation of control lymphocytes (Figure 4A, *pEGFP-N1 images* quantified in the histogram on the right*, 30% of cells*), as described previously [6]. When compared with EGFP-transfected cells, this gp120-induced CD4-CXCR4 capping was impaired in cells overexpressing functional gelsolin-EGFP (Figure 4A, *gelsolin-EGFP images* quantified in the histogram on the right, *20% of cells*) and similarly, rs-gp120 protein-induced CD4-CCR5 capping was blocked in permissive lymphocytes transiently transfected with gelsolin-EGFP (Figure 4B, *gelsolin-EGFP images* quantified in histogram*, 19% of cells*). These findings suggest that an increase in the actin-severing capacity induced by functional gelsolin overexpression provokes defective HIV-1 Env-gp120-mediated CD4-CXCR4 and -CCR5 capping at the cell surface, which may account for the blockade of HIV-1 infection by X4- and R5-tropic viruses, as described previously [6,7,9].

### Over-expression of gelsolin inhibits HIV-1 Env-mediated cell-to-cell fusion, regardless of viral tropism

**Figure 5:**
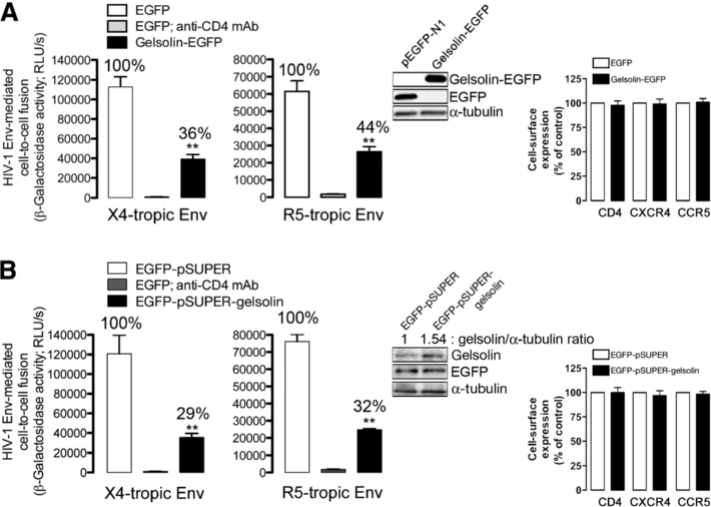
**Gelsolin functional overexpression impairs HIV-1 Env-gp120-mediated cell-to-cell fusion, regardless of viral Env tropism.** Effect of gelsolin-EGFP (**A**) or free gelsolin (**B**; *co-expressed with free EGFP using an EGFP-pSUPER-gelsolin plasmid*) overexpression in HeLa P5 cells on HIV-1 Env-mediated membrane fusion measured by β-Galactosidase production, and referred to the EGFP or EGFP-pSUPER control condition (100% of membrane fusion). Treated HeLa P5 cells were fused with HeLa cells expressing X4-tropic (243) or R5-tropic (ADA) Env, in the presence or absence of an anti-CD4 mAb. The data represent the mean ± s.e.m. of four independent experiments carried out in triplicate: ** p < 0.01, Student’s *t*-test. Western blot quantification of gelsolin-EGFP (**A**), free EGFP (**A** and **B**) and gelsolin (**B**; *detection of endogenous and overexpressedfree gelsolin*) expression together with total α-tubulin levels prior to cell-to-cell fusion, showing representative data for each experimental condition. Cell-surface expression of viral receptors was analyzed under each experimental condition (**A** and **B**). Data represent the mean ± s.e.m. of four independent experiments carried out in triplicate.

We assessed the effect of gelsolin activity on the gp120/gp41 fusion capacity in permissive HeLa P5 cells (Figure 5), in order to ascertain whether actin-severing gelsolin inhibits early HIV-1 infection during pore fusion formation or at a post-fusion step. In agreement with the previous experiments on permissive lymphocytes, the expression of CD4, CXCR4, and CCR5 was not modified by the overexpression of gelsolin-EGFP or free gelsolin, in these HeLa P5 cells (Figure 5A or B, respectively; *see cell-surface expression and Western blots*). Under these conditions, gelsolin-EGFP-transfected HeLa P5-permissive cells were much less susceptible to fuse with HeLa cells stably expressing X4- or R5-tropic Env (Figure 5A, *64% and 56% of inhibition, respectively*), compared to EGFP expressing cells (Figure 5A). Moreover, fusion with X4- and R5-tropic Env + cells was impaired in gelsolin-transfected HeLa P5 cells (Figure 5B, *71% and 68% of inhibition, respectively*). These results indicate that the efficiency of early HIV-1 infection is negatively regulated by overexpression of actin-severing gelsolin, which seems to act during the Env-induced virus/cell-membrane fusion step, constituting a process that appears to be directly related to the F-actin cortical content and dynamics.

### Specific RNA interference of endogenous gelsolin inhibits early HIV-1 infection and HIV-1 Env-mediated membrane fusion

**Figure 6:**
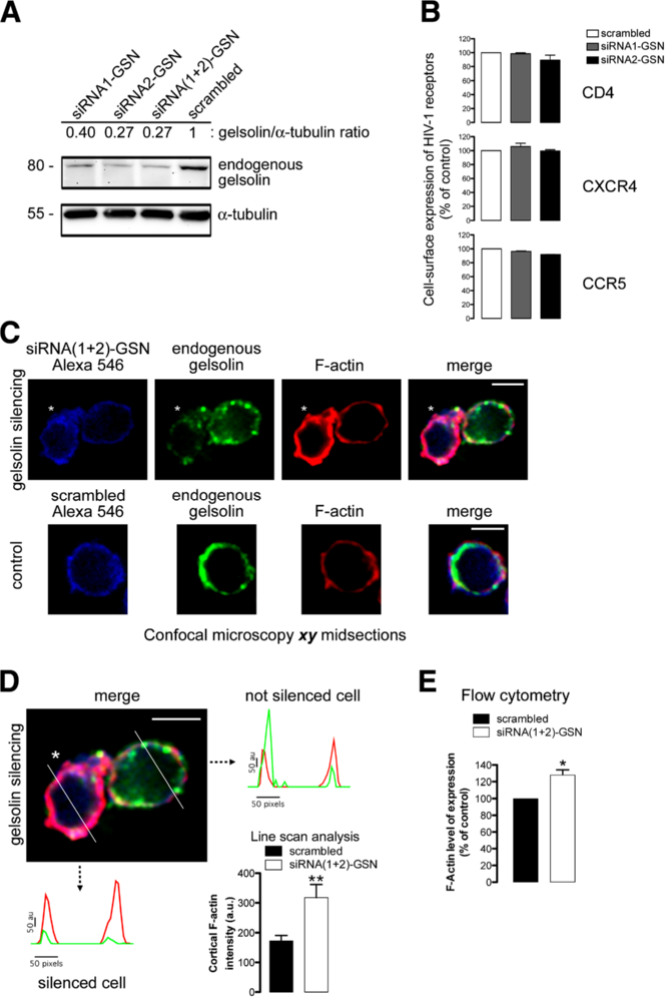
**Specific silencing of endogenous gelsolin and its effects on cortical F-actin, in permissive lymphocytes.** (**A**) Western blot of endogenous gelsolin knockdown in permissive lymphocytes transfected with two siRNA-GSN oligos, either individually (siRNA1-GSN or siRNA2-GSN) or combined (siRNA(1 + 2)-GSN), normalized to the expression of total α-tubulin. Cells transfected with scrambled siRNA were used as control. One representative experiment of three is shown. (**B**) Flow cytometry analysis of CD4, CXCR4 and CCR5 cell-surface expression in lymphocytes transfected with scrambled siRNA, siRNA1-GSN or siRNA2-GSN. (**C**) Confocal analysis of F-actin and endogenous gelsolin expression in siRNA(1 + 2)-GSN-treated lymphocytes, using a mixture of fluorescent Alexa-546 oligos. Fluorescent scrambled Alexa 546 was used as a control. White asterisks indicate cells in which endogenous gelsolin expression was efficiently silenced, expressing more cortical F-actin than in their neighbouring cells in which the uptake of siRNA(1 + 2)-GSN oligos was insufficient to silence gelsolin expression. ***xy*** midsections and merged images from a representative experiment are shown. Scale bar = 5 μm. (**D**) Line scans show fluorescence intensity profiles of cortical F-actin in silenced, siRNA(1 + 2)-GSN-treated cell and in the not silenced neighbour cell. Images correspond to the results presented in merged images in panel **B**, analysed along the horizontal lines drawn in panel **D**. Scale bar =5 μm. Histograms show the quantification of fluorescence intensity profiles of cortical F-actin in a series of cells (12 cells per series of siRNA(1 + 2)-GSN- and scrambled-treated cells) analysed by line scanning confocal microscopy (8 cortical points analyzed per cell). (**E**) Flow cytometry analysis of F-actin in scrambled- (control, taken as 100% F-actin expression) or siRNA(1 + 2)-GSN-treated cells. In **B** and **E**, data are mean ± s.e.m of three independent experiments carried out in triplicate. **p < 0.01, *p < 0.05, Student’s *t-*test.

**Figure 7:**
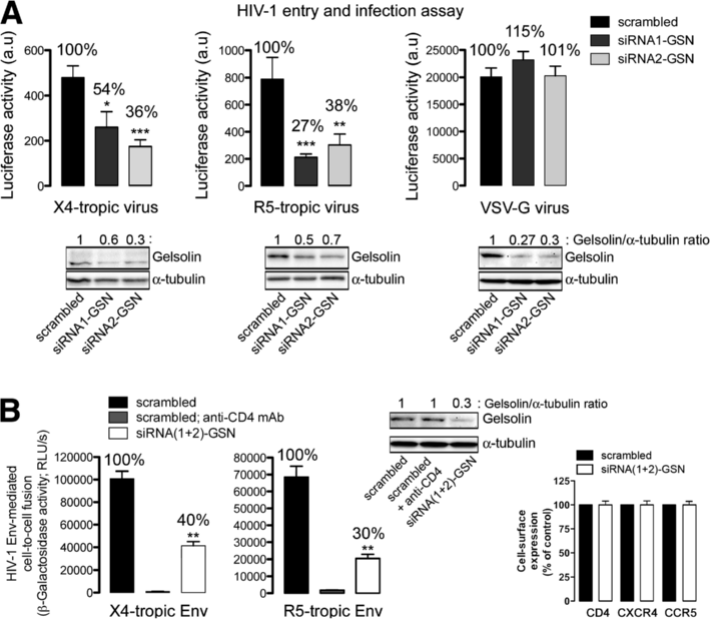
**Gelsolin knockdown restricts HIV-1 Env-mediated membrane fusion and infection in permissive cells, regardless viral tropism.** (**A**) Luciferase-based assay of viral entry and infection by non-replicative X4- and R5-tropic HIV-1 viral strains in siRNA1-GSN- or siRNA2-GSN-silenced permissive lymphocytes (viral entry and infection was considered 100% in cells transfected with scrambled siRNA, *i.e.,* controls). All the data were corrected using the values of non-productive infection (baseline) obtained in the presence of a neutralizing anti-CD4 mAb (5 μg/mL) under the same experimental conditions. A luciferase-based assay with equal inputs of pseudotyped VSV-G virions was performed to control the specificity of gelsolin knockdown-mediated effects on HIV-1 viral entry and infection, and by using specific siRNA1-GSN or siRNA2-GSN oligos. These data were corrected with non-productive baseline values obtained using non-infective virus-like particles lacking VSV-G Env under the same experimental conditions. Data are mean ± s.e.m. of three independent experiments carried out in triplicate. *Bottom*, Western blot of endogenous gelsolin knockdown prior to infection (*top,* infection histograms), showing representative data for each experimental condition. Cell-surface expression of viral receptors was analyzed under each experimental condition as shown in Figure 6B. (**B**) Effect of specific gelsolin silencing (siRNA(1 + 2)-GSN) in HeLa P5 cells on HIV-1 Env-mediated membrane fusion measured by β-Galactosidase production, and referred to the scrambled control condition (100% of membrane fusion). Treated HeLa P5 cells were fused with HeLa cells expressing X4-tropic (243) or R5-tropic (ADA) Env, in the presence or absence of an anti-CD4 mAb. Western blot of endogenous gelsolin knockdown prior to cell-to-cell fusion, showing representative data for each experimental condition. Cell-surface expression of viral receptors was analyzed under each experimental condition. Data are mean ± s.e.m. of four independent experiments carried out in triplicate * p < 0.05, ** p < 0.01, *** p < 0.001, Student’s *t*-test.

To further demonstrate the involvement of gelsolin in early HIV-1 infection, we studied the effects of silencing endogenous gelsolin expression in permissive lymphocytes with specific small interfering RNA (siRNA) oligonucleotides (oligos) against gelsolin (siRNA-GSN). Two different siRNAs were administered independently or in combination (siRNA1-GSN or siRNA2-GSN and siRNA(1 + 2)-GSN), which effectively silenced endogenous gelsolin expression (Figure 6A) without affecting the cell-surface expression of HIV-1 receptors (Figure 6B). Silencing endogenous gelsolin expression in permissive lymphocytes markedly enhanced the accumulation of cortical F-actin compared with non-interfered cells (asterisk in Figure 6C or D compared with neighbouring cell; *see line scan analysis*) or with control cells transfected with scrambled siRNA (*control* in Figure 6C, and Figure 6D, histograms line scan). These data were confirmed by quantifying total levels of F-actin expression by flow cytometry (Figure 6E), which revealed that gelsolin knockdown increases the levels of F-actin in CEM.NKR-CCR5 permissive lymphocytes. Moreover, silencing endogenous gelsolin expression negatively affected HIV-1 entry and infection in function of the degree to which endogenous gelsolin was silenced (Figure 7A). The inhibition of HIV-1 entry and infection again appeared to be independent of viral tropism as no differences were observed between gelsolin-silenced permissive lymphocytes infected with X4- or R5-tropic HIV-1 viral strains (Figure 7A).

The entry and infection of VSV-G pseudotyped viral particles, using equal viral inputs for these virions that for X4- and R5-tropic HIV-1 strains, were not affected by the specific silencing of endogenous gelsolin using siRNA1-GSN or siRNA2-GSN oligos (Figure 7A). Therefore, it appears that gelsolin silencing specifically affects HIV-1 Env-mediated viral entry and infection. However, we cannot fully rule out the involvement of gelsolin in endocytic VSV-G-driven viral entry, where it may affect specific actin structures associated with the clathrin-endocytic system. As such, further studies will be required to determine the role of the gelsolin/actin system during early VSV-G infection. Moreover, gelsolin knockdown (Figure 7B, *Western blot*) inhibits HIV-1 Env-mediated cell-to-cell fusion, regardless of HIV-1 Env tropism and without affecting HIV-1 cell-surface receptors on HeLa P5 permissive cells (Figure 7B). Of note, overexpression of EGFP or gelsolin-EGFP or nucleofection of scrambled or siRNA(1 + 2)-GSN oligos did not induce cellular toxicity in CEM-CCR5 or HeLa P5 permissive cells (Additional file 1).

Together, these findings suggest that endogenous gelsolin severs the cortical actin cytoskeleton to maintain the appropriate amount of F-actin that ensures correct dynamic actin reorganization during early HIV-1 entry and infection, and acting on the HIV-1 Env-induced virus/cell-membrane fusion process. Thus, alteration of endogenous gelsolin expression or activity, and subsequent cortical actin amount restrict early HIV-1 infection at a pre-fusion step.

### Knockdown of endogenous gelsolin impairs HIV-1 Env-gp120-mediated F-actin reorganization and viral receptor clustering

**Figure 8:**
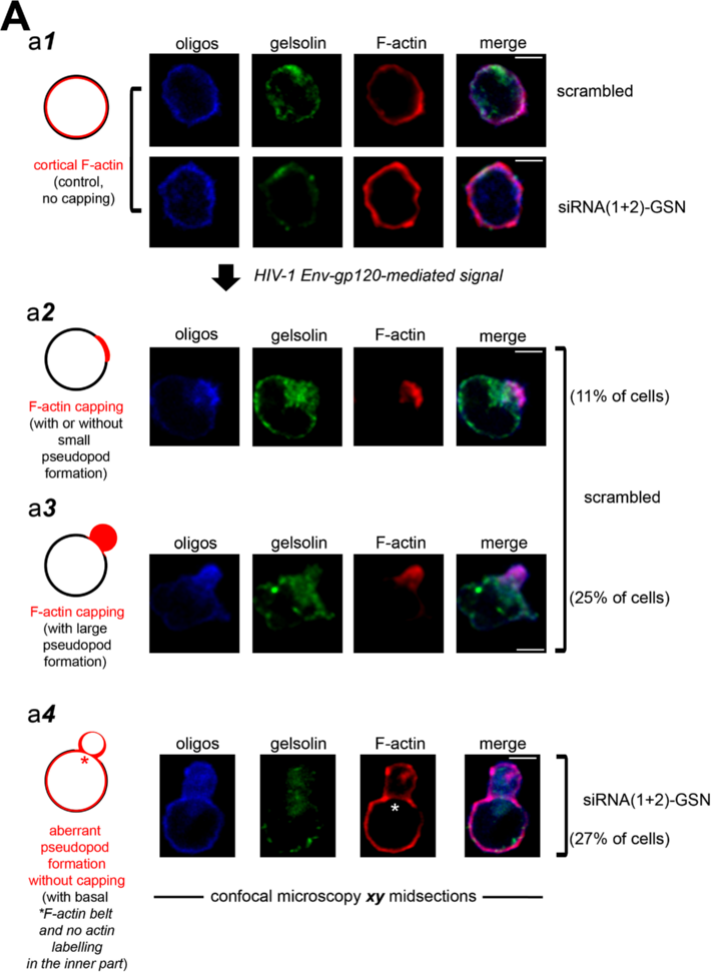
**Gelsolin controls HIV-1 Env-gp120-induced cortical actin reorganization and capping, and the changes in pseudopodia morphology.** (**A**) HIV-1 Env-gp120-induced cortical F-actin redistribution and cell morphologies of siRNA(1 + 2)-GSN-silenced lymphocytes, illustrated in the cartoons shown on the left (from *a****1*** to *a****4***). *a****1***, a series of confocal images showing the distribution of endogenous gelsolin and F-actin in the absence of any Env-gp120 stimulus (control, no capping) and in endogenous gelsolin-silenced cells suppressed using a combination of fluorescent siRNA(1 + 2)-GSN oligos. Scrambled fluorescent oligos were used to control for RNA interference. Cells lacking gelsolin accumulated more cortical actin than control cells. *a****2***, a series of confocal images shows HIV-1 Env-gp120-induced distribution of endogenous gelsolin and F-actin in control cells. An F-actin capping region was observed in 11% of the 200 cells counted, with a pseudopodium evident in some cases. *a****3***, a series of confocal images shows HIV-1 Env-gp120-induced formation of a pseudopodium containing the F-actin capping region, associated with gelsolin, in cells transfected with fluorescent scrambled siRNA. An F-actin capping region is visible within the prominent pseudopodium (25% of the 200 cells counted). *a****4***, a series of confocal images shows aberrant HIV-1 Env-gp120-induced pseudopodium formation and the absence of an F-actin capping region in siRNA(1 + 2)-GSN-silenced lymphocytes. An F-actin belt (asterisk) is visible at the base of the aberrant large pseudopodium, which contains no F-actin in its inner region (27% of the 200 siRNA(1 + 2)-GSN-silenced cells counted, where endogenous gelsolin levels were reduced together with an increase of cortical F-actin levels). This profile was not observed in control cells transfected with scrambled siRNA in any experimental condition. In all panels, scale bar = 5 μm and one representative experiment of three with merged images are shown. Scale bar = 5 μm.

To study the effect of gelsolin knockdown on the redistribution of cortical actin and the changes in cell morphology induced by HIV-1 Env-gp120, permissive lymphocytes transfected with siRNA(1 + 2)-GSN or with scrambled oligos were analysed by confocal microscopy (Figure 8). Cells exhibited a normal morphology and cortical F-actin distribution in the absence of stimulation by HIV-1 Env-gp120 (Figure 8A, panel a***1***) and in these conditions, transfection with siRNA(1 + 2)-GSN (fluorescent oligos) reduced the expression of endogenous gelsolin in conjunction with an increase in the amount of cortical F-actin (Figure 8A, panel a***1***). In permissive cells, cortical actin was redistributed to one of the poles of the cell when incubated with the rs-gp120_IIIB_ viral protein, forming a discrete cap of F-actin that in some cases morphologically resembled a small pseudopodium (Figure 8A, *panel* a***2***, *11% of cells*). Indeed, the majority of reactive control cells exhibited a prominent pseudopodium that developed in the region of the F-actin cap (Figure 8A, *panel* a***3***, *25% of cells*). These large pseudopodia containing reorganized cortical actin and they are indicative of efficient cellular activation by the viral Env-gp120 protein, as described previously [6]. While some endogenous gelsolin was detected within the F-actin capping region in all these cases (Figure 8A, *panels* a***2****and* a***3***, *36% of total capped cells*), it was not tightly restricted to the region of the actin cap, and endogenous gelsolin was also observed in the cytoplasm and at the plasma membrane of these activated cells (Figure 8A, *panels* a***2****and* a***3***). This event was also observed in HIV-1 Env-gp120-triggered permissive lymphocytes over-expressing free EGFP (Figure 3B, *EGFP-pSUPER (+) rs-gp120 series of images*). Remarkably, in cells transfected with siRNA(1 + 2)-GSN, redistribution of F-actin to one of the poles was not observed, and no pseudopodia were formed (similar as observed in panel a***1***, *siRNA(1 + 2)-GSN cells*). When gelsolin-silenced cells were stimulated with rs-gp120_IIIB_ pseudopodia with aberrant morphologies were formed in which no standard F-actin reorganization was observed (Figure 8A, *panel* a***4***, *27% of cells*). No F-actin was detected in the inner part of these aberrant pseudopodia, although F-actin did form a basal belt (Figure 8A, *panel* a***4***). Aberrant pseudopodia were not formed in cells transfected with scrambled oligos, which exhibited normal pseudopodium formation with an F-actin capping region (Figure 8A, *panel* a***3***, *25% of cells*).

**Figure 9:**
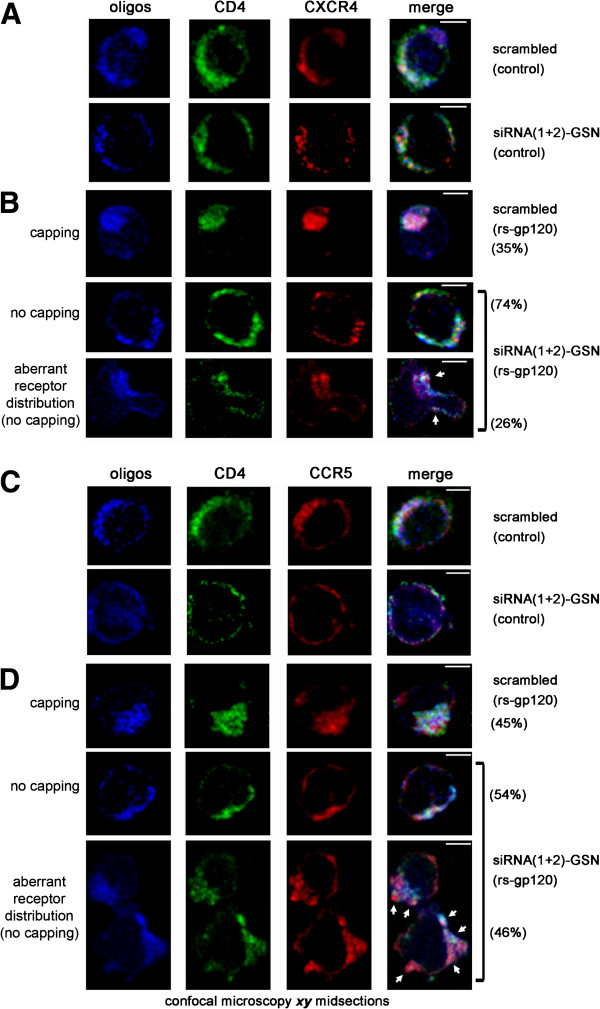
**Gelsolin knockdown inhibits the redistribution and clustering of CD4-CXCR4 and CD4-CCR5 induced by HIV-1 Env-gp120.** Confocal microscopy analysis of CD4 and CXCR4 (**A**) or CCR5 (**C**) distribution in control CEM.NKR-CCR5 cells transfected with fluorescent scrambled siRNA or siRNA(1 + 2)-GSN. Merged images show the co-localization of CD4-CXCR4 (**A**) or -CCR5 (**C**) at the plasma membrane. In (**B**) and (**D**), a series of ***xy*** midsections shows the HIV-1 Env-gp120-induced redistribution of CD4-CXCR4 and -CCR5, respectively, to one pole of the cell in CEM.NKR-CCR5 cells expressing scrambled Alexa 546 oligos 1 hour after exposure to rs-gp120_IIIB_ (5 μg/mL) at 37°C (capping, series of images). Merged images show cell surface CD4-CXCR4 (**B**) or -CCR5 (**D**) co-localization. In contrast, HIV-1 Env-gp120-induced CD4-CXCR4 and -CCR5 redistribution was not observed in cells transfected with siRNA(1 + 2)-GSN oligos (74% and 54% of cells showing no capping, respective series of images in (**B**) and (**D**)). These cells exhibited no CD4-CXCR4 or –CCR5 capping, although aberrant CD4 and CXCR4 or CCR5 redistribution and aberrant pseudopodium formation was evident (see white arrows in (**B**) or (**D**), respectively). The CD4-CXCR4 and -CCR5 clustering (35% and 45% of capping, respectively), the no capping and aberrant CD4-CXCR4 and -CCR5 distribution (26% and 46%, respectively) are reflected as a percentage of the 200 cells counted for each experimental condition, the data representing the results of three independent experiments. Scale bar = 5 μm. One representative experiment of three is shown per each experimental condition.

These results indicate that the actin-severing activity of endogenous gelsolin is required by HIV-1 to ensure normal actin dynamics and the appropriate amount of cortical actin accumulates at viral entry regions, where pseudopodia are subsequently formed. Accordingly, cells in which endogenous gelsolin is absent or reduced are refractory to HIV-1 entry and infection, due to alterations in these F-actin-dependent events. We and other groups demonstrated the importance of HIV-1-mediated cortical actin dynamics to promote the redistribution and clustering of CD4-CXCR4 and -CCR5 to one pole of the cell, events that enhance the initial cooperative virus-cell contacts, subsequent fusion pore formation, viral entry and infection [6-8]. Thus, we analyzed the effect of endogenous gelsolin knockdown on HIV-1 Env-gp120-induced-viral receptor redistribution and aggregation in permissive lymphocytes by confocal microscopy (Figure 9). The homogeneous distribution of CD4 and CXCR4 or CCR5 receptors on the plasma membrane of CEM.NKR-CCR5 permissive cells (Figure 9A and C, *scrambled (control)*, respectively) were rearranged in cells treated with the rs-gp120_IIIB_ viral protein (Figure 9B and D, *scrambled (rs-gp120)*, respectively). Thus, the CD4 and CXCR4 or CCR5 receptors redistributed to one pole of the activated cells, where they formed a large cluster or capping region (Figure 9B, *35% of cells*; and 9D, *45% of cells*). However, no receptor clustering was observed at the pole of cells transfected with gelsolin siRNA (Figure 9B and 9D, *siRNA(1 + 2)-GSN (rs-gp120)*), in which CD4 and CXCR4 or CCR5 were abnormally redistributed along aberrant pseudopodia (Figure 9B, *26% of cells*, and 9D, *46% of cells*, respectively) consistent with the F-actin labelling in similar experimental conditions (*see aberrant pseudopodium* in Figure 8A, *panel a****4***). This aberrant distribution of the cell-surface receptors required for HIV-1 infection, together with the impaired HIV-1 Env-gp120-mediated cortical actin reorganization and aberrant pseudopodium formation, might be responsible for the refractory behaviour of permissive lymphocytes lacking endogenous gelsolin.

Taken together, our data suggest that the actin-severing activity of gelsolin is fundamental for HIV-1 to influence cortical actin dynamics, and to appropriately adjust the amounts of actin available for reorganization during viral fusion, entry and infection. Moreover, we demonstrate that the influence of gelsolin on cortical actin affects HIV-1 Env-gp120 triggered CD4-CXCR4 or CD4-CCR5 clustering and HIV-1 Env-mediated cell-to-cell fusion, regardless viral Env tropism. Efficient HIV-1 fusion, entry and infection is therefore sensitive to the expression of gelsolin and its actin-severing activity. Hence, endogenous gelsolin can act as a limiting factor for early HIV-1 infection at a pre-fusion step.

## Discussion

In the present work, we analyzed the functional effect of cortical actin filament severing on early HIV-1 infection in permissive lymphocytes, and investigated whether gelsolin can compromise the viral-induced F-actin reorganization and viral receptor capping events required for HIV-1 fusion and infection. Specific binding of the HIV-1 Env-gp120 protein to CD4 initiates signalling via moesin [6] and filamin-A [7], resulting in actin-dependent receptor clustering (reviewed in [1-3]). The recruitment of sufficient receptor/co-receptor complexes via actin polymerization is required to initiate efficient viral fusion and entry. However, the highly dynamic network of cortical actin may form a barrier to fusion pore enlargement and the passage of the capsid into the cytoplasm [8,11,26]. Activation of cofilin, an actin depolymerization factor, is required to overcome the barrier generated by cortical actin in resting T cells, in a virus/cell post-fusion step [8]. Based on the promotion and impairment HIV-1 fusion and entry by moesin and cofilin or syntenin-1, respectively, cortical actin has been proposed to be dynamically and temporally remodelled by the virus itself during early HIV-1 infection [6,8,10]. Interference with this process inhibits HIV-1 viral fusion and infection, suggesting that dynamic intact actin is required for efficient viral fusion and infection (reviewed in [1,3]). Moreover, it is plausible that the action of other actin cytoskeleton-associated cellular components may be crucial for early HIV-1 entry and infection, as proposed here for gelsolin.

Our results indicate that overexpressed gelsolin co-localizes with cortical F-actin in the cytoplasm of permissive T lymphocytes. Gelsolin binds to actin filaments with high affinity (Kd of 50 nM) and it is thought to sever actin filaments (reviewed in [14]). After severing, gelsolin remains attached to the barbed ends of the filaments as a cap, thereby preventing the reannealing of actin fragments, and it constitutively co-localizes with the modified actin-filaments [21]. We found that overexpression of functional gelsolin diminished the amount of F-actin in permissive lymphocytes, which had a thinner F-actin cortex than the corresponding controls. In the cell cortex, gelsolin co-localized with F-actin without affecting the cell-surface expression of CD4, CXCR4 and CCR5 co-receptors, which appeared to be functional in our experimental conditions as they underwent normal ligand-stimulated internalization. By contrast, specific silencing of endogenous gelsolin expression increased the amount of cortical F-actin in the cell. Taken together, these results suggest that intracellular gelsolin severs or trims actin filaments in permissive T cells.

The studies of early HIV-1 infection by single-cycle viruses indicate that HIV-1 entry and infection is restricted when gelsolin is overexpressed. While HIV-1 Env-gp120 triggers cortical F-actin reorganization and polar clustering in permissive lymphocytes under control conditions, as described previously [6], the F-actin reorganization and localization at one pole of the cell induced by HIV-1 Env-gp120 was impaired in cells overexpressing functional gelsolin. Moreover, this effect was directly correlated with the inhibition of early HIV-1 infection in these cells, and with the inhibition of HIV-1 Env-mediated cell-to-cell fusion in HeLa permissive cells overexpressing gelsolin. Hence, our data indicate that cortical actin reorganization is fundamental for efficient viral fusion, entry and infection. The amount of actin available and its arrangement in the cortex appears to be controlled by the activity of the gelsolin expressed by the cell, suggesting that gelsolin can restrict early HIV-1 infection.

It is conceivable that the expression of gelsolin may increase in permissive cells in response to specific signals, resulting in altered actin-cytoskeleton dynamics due to the severing of cortical actin filaments (*i.e.,* bypassing the signals mediating by early HIV-1 infection). This activated cellular state may render target cells less permissive or refractory to HIV-1 infection *in vivo*. Indeed, gelsolin overexpression also inhibits HIV-1 Vpr-induced T-cell apoptosis by blocking the interaction between Vpr and the voltage-dependent anion channel (VDAC) [27], suggesting that gelsolin acts as a protective factor against Vpr-induced T-cell apoptosis. Activation of gelsolin’s basal actin-severing activity may also be important to facilitate the establishment of HIV-1 latency in infected cells, as suggested by the chemokine-induced, cofilin-dependent changes observed in the actin cytoskeleton of infected resting CD4+ T cells [28]. Further studies will be necessary to explore this hypothesis, and to elucidate the role of gelsolin in conditions of cell activation and infection.

The functional role of gelsolin-mediated actin severing during HIV-1 fusion and infection was further demonstrated in HIV-1 Env-mediated membrane fusion models and in infected permissive lymphocytes in which endogenous gelsolin expression had been silenced by specific RNA interference. The degree of gelsolin silencing is correlated with the extent to which HIV-1 entry and infection, and HIV-1 Env-induced cell-to-cell fusion were blocked in these cells. Specific gelsolin knockdown had no effect on CD4, CXCR4 and CCR5 cell surface expression and distribution in cells not stimulated with HIV-1 Env-gp120. Moreover, the inhibition of HIV-1 Env-mediated membrane fusion, viral entry and infection were independent of viral tropism. These findings were somewhat expected, as HIV-1 Env-mediated pore fusion formation and early viral infection are directly dependent on correct cortical actin dynamics and actin reorganization, events known to be crucial for successful infection with both tropic viruses [6-9].

Permissive lymphocytes transfected with gelsolin siRNA accumulated more cortical F-actin than control cells. Incubation with the HIV-1 Env-gp120 viral protein provokes a redistribution of cortical actin to one pole of control permissive cells, where it forms a discrete F-actin capping region within small or large pseudopodia. These changes in cell morphology and actin dynamics are indicative of efficient cellular activation by the viral Env-gp120 protein [6,10]. In such cases, a proportion of the endogenous gelsolin was distributed within the F-actin capping region, but without displaying a clear-cut capping pattern. By contrast, no redistribution of F-actin nor capping was mediated by the viral protein in cells transfected with gelsolin siRNA, which also exhibited aberrant pseudopodia formation. Aberrant pseudopodia were characterized by the absence of detectable F-actin in their interior and the presence of an F-actin belt at the base of the pseudopodium. Significantly, alterations in the morphology and retractile properties of actin-dependent filopodia have been described in cells from gelsolin null mice [17].

Taken together, these data indicate that the actin-severing activity of endogenous gelsolin is required by HIV-1 to ensure that Env-gp120 induces a redistribution of cortical actin, appropriate actin dynamics and the accumulation of the necessary amount of cortical actin at the viral-cell contact and entry regions, where pseudopodia subsequently form. Accordingly, cells in which endogenous gelsolin expression is reduced or absent are refractory to HIV-1 Env-mediated membrane fusion, viral entry and infection due to impairment of these multiple F-actin-driven events.

Gelsolin-mediated F-actin-severing occurs in the presence of full-length intracellular or recombinant gelsolin [14] and the severing of F-actin occurs only when a sufficient number of actin-actin bonds are broken [29]. Specific cell signalling events can lead to the formation of plasma membrane-associated structures (*e.g.,* lamellipodia, pseudopodia and filopodia), a process that is regulated by gelsolin and other related tropomyosins through the rearrangement of actin filaments [17,18,30-34]. The actin-severing activity of gelsolin can rapidly restructure the cytoskeleton. In addition, differences in cortical actin content between resting memory and naive T cells are likely to contribute to their differential susceptibility to HIV-1, which is enhanced in resting T cells [35]. Taken together with the present findings, these observations suggest that HIV-1 requires a minimum level of gelsolin to drive the restructuring of the cortical actin cytoskeleton in the areas of viral-cell contact and viral entry, thereby ensuring efficient HIV-1 Env-induced pore fusion formation and subsequent viral entry and infection.

Our results also suggest that gelsolin mediates virus-triggered receptor clustering by modulating HIV-1 Env-gp120-mediated cortical actin dynamics. The viral protein induces a redistribution of cell-surface CD4 and CXCR4 or CCR5 receptors to one pole of activated control cells, an effect that is abrogated by gelsolin overexpression or knockdown. Indeed, cells in which gelsolin expression was silenced exhibited alterations in the redistribution of CD4 and CXCR4 or CCR5 in aberrant pseudopodia, similar to the redistribution of F-actin in equivalent circumstances. This inhibition of virus-induced receptor clustering [6,7] may account for the observed inhibition of HIV-1 Env-mediated membrane fusion and early viral infection following the silencing of gelsolin expression.

The aforementioned observations suggest that gelsolin severs the cortical actin cytoskeleton to maintain its proper architecture with the appropriate amount of F-actin, ensuring its dynamic reorganization during early HIV-1 entry and infection. As a consequence of this dynamic reorganization of F-actin, receptors mediating viral infection are driven to one pole of the cell where they co-localize. Perturbing these actin- and gelsolin-mediated events severely restricts pore fusion formation and early HIV-1 infection, which are therefore sensitive to the actin-severing activity of gelsolin at the plasma membrane, and hence, to gelsolin expression. Accordingly, endogenous gelsolin may act as a limiting factor for early HIV-1 entry and infection. This hypothesis was validated by analyzing the effects of gelsolin overexpression or specific knockdown on early infection with endocytotic VSV-G pseudotyped viral particles. The impaired infectivity of HIV-1 in gelsolin overexpressing lymphocytes, or in gelsolin-silenced cells were complemented with VSV-G pseudotyped virions, which fuse in low-pH endocytotic vesicles. This is consistent with the fact that HIV-1 mainly fuses and infects T lymphocytes by exploiting both actin-dependent and Arf6 GTPase-regulated, clathrin-independent, plasma membrane dynamic pathways [6-9,24,36]. However, we cannot rule out the involvement of gelsolin in the clathrin-endocytic VSV-G-driven viral entry route after activation of specific cellular or signalling processes.

In fact, considering all the above data and discussion, we suggest that efficient early HIV-1 infection requires the appropriate amount of cortical actin to be reorganized in a specific manner during the initial virus-cell contacts. Both these processes appear to be influenced by actin-severing gelsolin, suggesting that alteration of gelsolin activity or expression can restrict HIV-1 entry and infection. Therefore, both overexpression of functional gelsolin or specific knockdown of endogenous gelsolin negatively affects HIV-1 Env-mediated actin dynamics, entry and infection at a pre-fusion step. Cofilin regulates early HIV-1 infection depolymerising cortical F-actin at a post-fusion step [8], a process that may be influenced by cortical F-actin levels, in cells where endogenous gelsolin was silenced. It is plausible that cofilin found difficulties to inhibit HIV-1 infection in cells presenting higher amounts of cortical F-actin, after knockdown of endogenous gelsolin. Moreover, gelsolin silencing impairs early HIV-1 infection acting in a pre-fusion step, thereby preceding, and maybe abrogating cofilin-mediated post-fusion effects on HIV-1 infection. The fact that silencing of gelsolin expression does not completely inhibit HIV infection could be due to the existence of cells where siRNA-GSN oligos did not correctly silenced endogenous gelsolin, as observed in cells from new Figure 6C or D, or to the presence of other actin-associated proteins or adaptors, such as moesin and filamin-A [6,7], which could compensate the negative effect of gelsolin knockdown.

These findings provide important insights into the complex molecular and actin-associated dynamics events that underlie early viral infection. Thus, we propose that gelsolin is a new factor that can limit HIV-1 infection acting at a pre-fusion step. Accordingly, cell-signals that regulate gelsolin expression and/or its actin-severing activity may be crucial to combat HIV-1 infection.

In this matter, cell signals that upregulate or downregulate the expression of functional gelsolin, or that alter its cortical localization, may render putative target cells refractory to HIV-1 infection *in vivo*. Virus-mediated cell signalling may represent a potential therapeutic target to treat early HIV-1 infection (reviewed in [37]) and in fact, HIV-1 Env-fusogenic activity appears to be both Ca^2+^[38] and phosphatidylinositol (4,5)-bisphosphate (PIP_2_) dependent [10,22,24,36]. By activating type Iα phosphatidylinositol-4-phosphate 5-kinase (PI4P5-K Iα) at virus-cell contact regions, HIV-1 is known to trigger the production of this PIP_2_-fusogenic lipid to promote fusion pore formation and viral infection [22], and the depletion of syntenin-1 allows a higher pool of free PIP_2_ to accumulate at the plasma membrane after HIV-1 attachment [10]. Furthermore, PIP_2_-associated plasma membrane changes driven by Arf6 are crucial for early HIV-1 infection, ensuring cell surface regeneration when the virus fuses with and enters permissive cells [24,36,39]. It should be noted that gelsolin is regulated by both PIP_2_ and intracellular Ca^2+^, which may represent additional control points for actin dynamics in the plasma membrane [20,32,40-43]. Gelsolin-mediated F-actin reorganization may also drive the final stages of virus-cell lipid mixing, favouring viral fusion at the molecular level [44]. Given the proposed interplay between gelsolin, Ca^2+^ and PIP_2_, and the effect of gelsolin on early HIV-1 infection described here, better understanding the regulation of intracellular gelsolin localization and activation will be important to develop strategies to control HIV-1 infection of T lymphocytes.

## Conclusions

Our findings suggest that by severing cortical F-actin, gelsolin regulates actin dynamics, the amount of F-actin available for reorganization, lymphocyte plasma membrane morphology, and viral receptor capping during HIV-1 viral entry and infection. The fine-tuning of gelsolin activity is necessary to ensure the virus-driven regulation of cell-surface receptors and of cortical actin dynamics, events that are crucial for efficient pore fusion formation and therefore early HIV-1 infection in T lymphocytes. Thus, it appears that endogenous gelsolin maintains cortical actin dynamic during early viral infection, acting in a pre-fusion step. Hence, alteration of gelsolin expression or activity accounts for an abnormal HIV-1 Env-mediated cortical actin reorganization that negatively affects early viral infection. These data enhance our understanding of the complex molecular events that underlie early viral infection, and highlight the possibility of modifying gelsolin-mediated actin-severing to present a barrier to HIV-1 infection, regardless of viral tropism, during the first virus-cell contacts and membrane fusion events. Further studies will be necessary to elucidate the cell signals that up or downregulate intracellular gelsolin expression, or that modulate its actin-severing activity, thereby altering F-actin remodelling. Identifying such signals will also help further characterize the role of gelsolin in HIV-1 fusion, entry and infection of CD4+ T lymphocytes *in vivo*.

However, based on our results we propose that the actin-severing gelsolin protein is a novel limiting factor that controls early HIV-1 infection in permissive lymphocytes acting at a pre-fusion step.

## Methods

### Antibodies and reagents

The commercial mouse anti-gelsolin monoclonal antibody (mAb, clone GS-2C4; Sigma, St. Louis, MO) was used here. The neutralizing mAb RPA-T4 (eBioscience, San Diego, CA) directed against CD4 was either phycoerythrin (PE)-labelled (flow cytometry; confocal analysis) or left unconjugated (inhibition of HIV-1 entry and infection for confocal analysis). PE conjugates of the CD184 (clone 12G5) and CD195 (clone 2D7/CCR5) (BD Bioscience/BD PharMingen, San Jose, CA) are directed against the second extracellular loop of CXCR4 and CCR5, respectively. Biotinylated mouse anti-human CXCR4 and CCR5 mAbs were purchased from BD PharMingen. The rabbit anti-EGFP polyclonal antiserum (pAb) was obtained from Santa Cruz Biotechnology (Santa Cruz, CA), and the monoclonal anti-α-tubulin from Sigma-Aldrich (Sigma-Aldrich, St. Louis, USA). Secondary horseradish peroxidase (HRP)-conjugated antibodies were purchased from Dako (Glostrup, Denmark), while Alexa Fluor 568-phalloidin (to label F-actin), Alexa Fluor 488-goat anti-mouse, Alexa Fluor 633-goat anti-mouse and NeutrAvidin Texas Red conjugate were purchased from Molecular Probes (Eugene, OR). Endotoxin-free, recombinant soluble (rs) X4-tropic HIV-1 Env-gp120-IIIB protein (rs-gp120_IIIB_) was produced in *Escherichia coli* by Innogenetics (Ghent, Belgium), and used as described previously [6,22]. Stromal cell-derived factor (SDF)-1α (CXCL12) was kindly synthesized and provided by Francoise Baleux (Institut Pasteur, Paris, France) [24,45,46].

### Cells

The human CEM.NKR-CCR5 permissive cell line (catalog number 4376, NIH AIDS Research and Reference Reagent Program) was grown at 37°C in a humidified atmosphere with 5% CO_2_ in RPMI 1640 medium (Lonza, Verviers, Belgium) supplemented with 10% foetal calf serum (Lonza), 1% L-glutamine, and 1% penicillin-streptomycin. Cells were passaged every 3 days. The HeLa-P5 cells, constitutively expressing CXCR4 and stably transfected with human CD4 and CCR5 cDNAs and with an HIV-long terminal repeat-driven-β-galactosidase reporter gene [23,45,47], as well as, HeLa-243 and HeLa-ADA cells, co-expressing the Tat and X4- and R5-tropic HIV- 1-Env proteins, respectively, were provided by Dr. M. Alizon (Hôpital Cochin, Paris, France) and cultured as previously described [23,45,47]. The 293T cell line was similarly cultured in supplemented DMEM (Lonza). Cells were harvested and cultured to 50-70% confluence in fresh supplemented DMEM 24 hours before cell transfection with viral or human DNA constructs.

### Western blotting

Protein expression was determined in Western blots of cell lysates. Briefly, 24 h after nucleofection of cells with different cDNA constructs (pEGFP-N1, gelsolin-EGFP, EGFP-pSUPER or EGFP-pSUPER-gelsolin) or short interference oligonucleotides (oligos) using specific Amaxa kits (Amaxa, Köln, Germany), cells were lysed at 4°C in 1% Triton-X100 sample buffer supplemented with a protease inhibitor cocktail (Roche Diagnostics GmbH, Mannheim, Germany). Equivalent amounts of protein, as determined using the bicinchoninic acid method, were separated by SDS-PAGE on 10% gradient gels and electroblotted onto 0.45 μm polyvinylidene difluoride membranes (PVDF; Millipore Corporation, Billerica, MA) using Trans-blot Turbo (Bio-Rad, Hercules, CA). Membranes were then probed with specific antibodies and the proteins recognised were detected by luminescence using the ECL System (Pierce), and analyzed using a ChemiDoc MP device and Image LabTM Software, Version 4.0.1 (Bio-Rad).

### Messenger RNA silencing

We designed the following short interference oligos specifically directed against the indicated mRNA sequences of gelsolin (GSN): siRNA1-GSN (position 611–629; sense: 5^′^-acagcaaucgguaugaaag-3^′^-dTdT; antisense: 5^′^-cuuucauaccgauugcugu-3^′^-dTdT) and siRNA2-GSN (position 1692–1710; sense: 5^′^-cgaugccuuuguucugaaa-3^′^-dTdT; antisense: 5^′^-uuucagaacaaaggcaucg-3^′^-dTdT). Alexa 546-conjugated or non-fluorescent siRNA oligos, and scrambled control siRNA oligos (Alexa 546-conjugated or not), were obtained from Sigma-Aldrich. For confocal microscopy experiments, we used a mixture of both Alexa 546-conjugated siRNA-GSN oligos (siRNA(1 + 2)-GSN) to induce specific siRNA-mediated silencing of endogenous gelsolin expression. Permissive CEM.NKR-CCR5 cells were nucleofected with 1 μM siRNA-GSN or a commercial scrambled control siRNA using specific Amaxa kits (Amaxa), and the cells were assayed 24 h later, as described previously [6,22,24,48]. The siRNA for gelsolin induced specific interference of protein expression for at least 72 h, as witnessed in Western blots (data not shown). Cells nucleofected with scrambled or specific siRNA oligos against gelsolin were lysed and analysed with specific antibodies in Western blots to determine the endogenous gelsolin silencing.

### Viral DNA constructs and plasmids

The pNL4-3.Luc.R-E- provirus (catalog number 6070013), the X4-tropic HXB2-*envelope* (*env*, catalogue number 5040154) and R5-tropic pCAGGS-SF162-gp160-*env* (catalogue number 3041817) glycoprotein vectors, and the pHEF-VSV-G vector (catalogue number 4693) encoding the vesicular stomatitis virus G (VSV-G) protein, were obtained via the NIH AIDS Research and Reference Reagent Programme. For gelsolin-EGFP expression, total RNA from CEM.NKR-CCR5 T-cells was extracted, transcribed to cDNA, and a gelsolin construct lacking the stop codon was generated by Expand High Fidelity PCR using cDNA as a template and 5^′^-GGAATTCGCCACCATGGTGGTGGAACACCCCGAGTTCC-3^′^ (sense) and 5^′^-CGGGATCCCTGGCAGCCAGCTCAGCCATGGCCC-3^′^ (antisense) primers. The amplified gelsolin product was cloned into pEGFP-N1 (Clontech, Palo Alto, CA) between the EcoRI and BamHI restriction sites (Takara Bio Inc., Japan). Control pEGFP-N1 (Clontech) was used to express free EGFP in permissive lymphocytes.

To create a system which allowed us to express both free EGFP and gelsolin proteins, we cloned the human gelsolin coding sequence into pSUPER.retro.neo + GFP (Oligoengine, WA). This plasmid was digested with HindIII and BglII restriction enzymes (New England Biolabs, MA) and gelsolin was amplified from the GSN.EGFP construct used in this work and by using the following primers containing either a 5^′^ HindIII or BglII restriction sequence: 5^′^-GAGATCTGCCACCATGGTGGTGGAACACCCCGAGTTCC-3^′^ 5^′^-CGAAGCTTCTTCAGGCAGCCAGCTCAGCCATGGCCC-3^′^.

Polymerase Chain Reaction (PCR) was performed by using Phire Hot Start II DNA Polymerase (ThermoScientific, MA) and amplification conditions were such as 98°C/30sec; 35 cycles of 98°C/5sec, 68°C/5sec, 72°C/30sec; and 72°C/1min. The gelsolin amplicon was also digested with both HindIII and BglII, consecutively cloned into the HindIII/BglII site on pSUPER.retro.neo + GFP (named in this work as EGFP-pSUPER-gelsolin) and positive clones were selected through Ampicilin resistance. Control pSUPER.retro.neo + GFP plasmid is used as a control for expression of free EGFP (named EGFP-pSUPER in this work).

### Production of viral particles

Replication-deficient luciferase-HIV-1 or -pseudotyped VSV-G viral particles were obtained as described previously [6,22-24]. Briefly, replication-deficient viral particles were derived from the luciferase-expressing reporter virus HIV/Δ*nef*/Δ*env*/*luc* + (in which the luciferase gene is inserted into the nef ORF and does not express the envelope glycoprotein) with an X4-tropic (Lai), R5-tropic (SF162) or VSV-G Env glycoprotein. X4- or R5-tropic HIV-1 or pseudotyped VSV-G viral particles were produced in 12-wells plates by co-transfecting 293T cells (70% confluence) with pNL4-3.Luc.R-E- (10 μg) and X4-tropic (HXB2-env) or R5-tropic (pCAGGS SF162 gp160) or VSV-G (pHEF-VSV-G) Env glycoprotein vector (10 μg). Viral plasmids were transduced in 293T cells using X-tremeGENE HP DNA transfection reagent (Roche Diagnostics). After the addition of X-tremeGENE HP to the viral plasmids (at a plasmid:X-tremeGENE HP ratio of 1:3 [wt/v], for pNL4-3/R5, pNL4-3/X4 and pNL4-3/VSV-G viral vector combinations), the solution was mixed in 100 μL of DMEM medium (without serum or antibiotics) and incubated for 15 min at room temperature prior to adding it to 293T cells. The cells were cultured for 48 h to allow viral production. The viruses were harvested and the supernatant was clarified by centrifugation at 3000 x g for 30 min, filtered (0.45 μm pore size) and concentrated using Amicon Ultra-4 Centrifugal filter devices (Millipore). Virions were then stored at −80°C. Viral stocks were normalized by p24-Gag content, determined using an enzyme-linked immunosorbent assay (GenscreenTM HIV-1 Ag Assay; Bio-Rad, Marnes-la-Coquette, France).

### Luciferase viral entry and infection assay

Untreated or nucleofected CEM.NKR-CCR5 cells (9 × 10^5^ cells in 24-well plates with 20 μg/mL of polybrene) were infected for 2 h with a synchronous dose of luciferase-based X4- or R5-tropic HIV-1 or pseudotyped VSV-G viral inputs (500 ng of p24), in a total volume of 1 mL RPMI 1640 (by centrifugation at 1,200 g at 25°C), and again for 4 h at 37°C, as described previously [6,22-24]. Unbound virus was then removed by washing the infected cells and 48 h after infection, luciferase activity was measured using a luciferase assay kit (Biotium, Hayward, CA) and a microplate reader (VictorTM X5, PerkinElmer, Waltham, MA). Where indicated, permissive cells were pretreated with an anti-CD4 neutralizing mAb (5 μg/mL). The data were analyzed using GraphPad Prism 5.0 software (GraphPad Software, San Diego, CA) and the protein content was measured using the bicinchoninic acid method.

### HIV-1-Env-mediated cell-to-cell fusion assay

A β-Galactosidase cell fusion assay was performed as previously described [23,45,47]. Briefly, HeLa-243 or HeLa ADA cells were mixed with previously treated HeLa-P5 cells (*overexpressing free EGFP, gelsolin-EGFP, free gelsolin, scrambled or siRNA(1 + 2)-GSN oligos*), in 96-well plates, in a 1:1 ratio (20,000 total cells), under each experimental condition. These co-cultures were kept at fusion for 16 h at 37°C. The fused cells were washed with Hanks’ balanced salt solution, lysed, and the enzymatic activity was evaluated by chemiluminescence (β-Galactosidase reporter gene assay; Roche Diagnostics, Germany). Anti-CD4 neutralizing mAb (5 μg/ml was preincubated in HeLa-P5 cells for 30 min at 37°C before co-culture with Env + HeLa cells (243 or ADA) was used as a control for the blockage of cell fusion.

### Immunofluorescence

Transfected immunofluorescent CEM.NKR-CCR5 (1 × 10^6^ cell/point) cells were washed three times with PBS and fixed for 5 min in 3% paraformaldehyde in PBS. The cells were again washed three times with PBS and where indicated, permeabilized with 0.1% Triton X-100 in PBS. After washing with PBS, the cells were immunostained for 1 h at room temperature with Alexa 568-labeled phalloidin, Alexa 633-labeled goat anti-mouse against CD4 (previously incubated with a specific mAb), or NeutrAvidin Texas Red conjugate against anti-CXCR4- or anti-CCR5-biotinylated Abs (previously incubated with a primary specific mAb against viral co-receptors), all diluted in PBS containing 0.1% BSA. Coverslips were mounted in Mowiol-antifade (Dako) and imaged in ***xy*** midsections with a FluoView FV1000 confocal microscope using a 1.35 NA objective (60x; Olympus, Center Valley, PA) for high-resolution imaging of fixed cells. The final images were analyzed and quantified with MetaMorph software (Universal Imaging, Downington, PA). Line scan analysis of the expression and distribution of cortical F-actin in gelsolin-EGFP, free gelsolin (co-expressed with free EGFP using a pSUPER plasmid) and free EGFP overexpressing cells was quantified using MetaMorph software, as described previously [48].

### CD4-CXCR4 and CD4-CCR5 clustering assay

Viral receptor capping assays were performed as described previously [6,49]. Briefly, CEM.NKR-CCR5 permissive cells expressing CD4, CXCR4 and CCR5 were co-cultured with the HIV-1 Env-gp120 protein rs-gp120_IIIB_ (5 μg/ml) for 1 hour at 37°C. The viral Env-gp120 protein was then washed out and the cells were fixed with 3% paraformaldehyde. The distribution of CD4 and CXCR4 or CD4 and CCR5 at the cell-surface of gelsolin-EGFP or siRNA(1 + 2)-GSN Alexa 546 expressing cells (and their corresponding controls) was determined by immunofluorescence and analyzed by confocal microscopy, as described above. The final images were analyzed and quantified with MetaMorph software.

### F-actin capping assay

Confocal analysis of HIV-1 Env-gp120-induced F-actin redistribution was performed in permissive lymphocytes as described previously [6]. Cells in which endogenous gelsolin was previously knocked-down by transfection with fluorescent siRNA(1 + 2)-GSN Alexa 546 (scrambled Alexa 546 oligos were used as a control), or those in which gelsolin-EGFP or free gelsolin (co-expressed with free EGFP using a pSUPER plasmid) was overexpressed (overexpression of free EGFP was used as a control, in both experimental conditions) were co-cultured with the HIV-1 Env-gp120 protein rs-gp120_IIIB_ (5 μg/ml) for 1 hour at 37°C. The viral Env-gp120 protein was then washed out and the cells were fixed with 3% paraformaldehyde. The cells were washed three times with PBS after fixation and then permeabilized with 0.1% Triton X-100 in PBS. The cells were subsequently washed with PBS after permeabilization and immunostained for 1 h at room temperature with Alexa 568-labeled phalloidin. The siRNA(1 + 2)-GSN Alexa 546-treated cells were also immunostained with an Alexa 488-conjugated goat anti-mouse antibody against endogenous gelsolin or Alexa 633-conjugated anti-gelsolin Ab in the case of cells expressing free EGFP. Cells were analyzed by confocal microscopy, as described above, to obtain high-resolution images of the fixed, permeabilized cells. The final images were analyzed and quantified using MetaMorph software.

### Flow cytometry analysis

For cell-surface analysis of viral receptors, CEM.NKR-CCR5 and HeLa P5 cells (3 × 10^6^ cell/point) nucleofected with different DNA constructs (pEGFP-N1, 0.5 μg; gelsolin-EGFP, 3 μg; EGFP-pSUPER or EGFP-pSUPER-gelsolin, 0.5 or 3 μg, respectively; or the RNA interfering oligos siRNA(1 + 2)-GSN or scrambled siRNA, 1 μM) were incubated with PE-labelled specific antibodies against CD4, CXCR4 or CCR5 (1/20 dilution) for 1 h at 4°C to prevent receptor internalization. Next, the cells were washed with ice-cold PBS, fixed in PBS containing 3% paraformaldehyde and analyzed by flow cytometry (XL-MCL System, Beckman-Coulter Inc.). For intracellular analysis of cortical F-actin, permissive CEM.NKR-CCR5 cells (3 × 10^6^ cell/point) nucleofected with the different DNA constructs (pEGFP-N1, 0.5 μg; gelsolin-EGFP, 3 μg; or the RNA interfering oligos siRNA(1 + 2)-GSN or scrambled siRNA, 1 μM) were fixed in PBS containing 3% formaldehyde and then permeabilized in 0.1% Triton X-100 buffer. The cells were then incubated with Alexa 568-labeled phalloidin for 1 h at room temperature, washed with PBS, fixed in PBS containing 3% paraformaldehyde and finally analyzed by flow cytometry (XL-MCL system, Beckman-Coulter Inc.).

### Flow cytometry analysis of cellular F-actin content

The intracellular actin content was analyzed as described previously [6]. Briefly, 24 h after transfection with the different DNA constructs (pEGFP-N1, 0.5 μg; gelsolin-EGFP, 3 μg), or with scrambled or siRNA(1 + 2)-GSN oligos (1 μM), permissive lymphocytes were fixed to stop intracellular actin dynamics with a solution containing 3% formaldehyde in PBS, 1% Triton X-100, and 5 μg/ml Alexa Fluor 568-phalloidin. Cells were incubated for 15 minutes at 37°C and washed once in PBS and the levels of intracellular polymerized actin (F-actin) were determined using a FACScan flow cytometer (XL-MCL System, Beckman-Coulter Inc.).

### Ligand-induced CXCR4 endocytosis

CEM.NKR-CCR5 cells (0.5 × 10^6^ cells/mL) were incubated with either 50 or 200 nM of SDF-1α for 1 h at 37°C in serum-free RPMI 1640, as described previously [23,24,46]. The cells were then incubated for 3 min at 4°C in an acidic buffer (50 mM glycine [pH 3.0]), to stop receptor endocytosis and remove CXCR4-bound SDF-1α that masks cell-surface CXCR4 receptor recognition by specific mAbs. The cells were then washed twice with ice-cold PBS and 0.1% BSA, incubated with specific anti-receptor PE-conjugated mAbs (1:50) for 1 h at 4°C, and then fixed with 3% paraformaldehyde in PBS. Samples were analyzed on a flow cytometer (XL-MCL System) to measure cell-surface receptor labelling in free EGFP + and gelsolin-EGFP + cells. Basal cell fluorescence intensity for cell-surface CXCR4 was determined by staining cells with a PE-conjugated IgG2a isotype control.

## Abbreviations

(HIV-1): Human immunodeficiency virus type 1; (Env): Envelope; (rs-gp120): Recombinant soluble HIV-1 Env-gp120 viral protein; (siRNA): Small interfering RNA; (oligos): Oligonucleotides; (VSV-G): Vesicular stomatitis virus-G Env protein; (GSN): Gelsolin; (GTPases): Guanosine-triphosphatases; (EGFP): Enhanced green fluorescent protein.

## Competing interests

The authors declare that they have no competing interests.

## Authors’ contributions

LG-E, SZ, JB-G, LA-R and M-SV performed the experiments and contributed to data acquisition. LG-E, SZ, JB-G, LA-R, M-SV, DZ, J-DM and AV-F analyzed the data. AV-F conceived and designed the study and experiments, and wrote the paper. All authors have read and approved the final manuscript.

## Supplementary Material

Additional file 1 **Flow cytometry analysis of cell viability of permissive cells, under different experimental conditions.** Description of data: (A) Flow cytometry-based analysis of propidium iodide uptake (FL-3) in pcDNA.3-, EGFP- and gelsolin-EGFP-transfected permissive cells (top panel, permissive lymphocyte cells; bottom panel, permissive HeLa cells) at 24 h post-transfection. Expression of EGFP- or gelsolin-EGFP is monitored in FL-1. Quantification of propidium iodide uptake (FL-3) by these treated cells (FL-1) is indicated in regions R of plots (represented as the percentage of total cells analyzed), per each experimental condition. A representative experiment of three is shown. (B) Flow cytometry-based analysis of propidium iodide uptake (FL-3) in scrambled and siRNA(1 + 2)-GSN-treated permissive cells (top panel, permissive lymphocyte cells; bottom panel, permissive HeLa cells) at 24 h post-nucleofection. Quantification of propidium iodide uptake (FL-3) by these treated cells (SSC, Side Scatter) is indicated in regions R of plots, per each experimental condition. A representative experiment of three is shown. In (A) and (B), not any significant toxicity is observed under each experimental condition. Click here for file
