# Supplementary figures and images for: Gelsolin activity controls efficient early HIV-1 infection
Source: Retrovirology. 2013 Apr 10;10:39. doi: 10.1186/1742-4690-10-39 (PMC3626799; doi:10.1186/1742-4690-10-39)

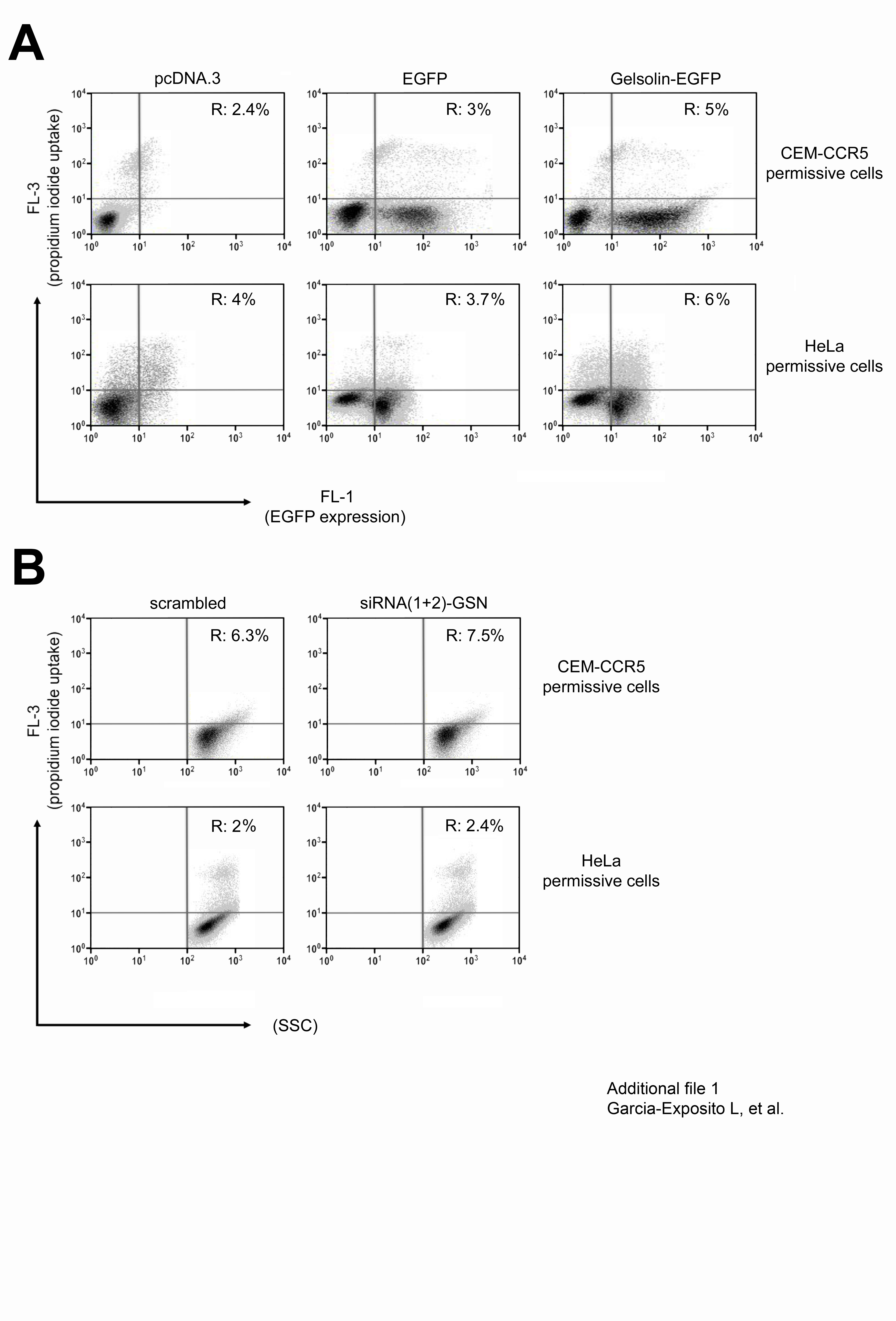

Supplement: Additional file 1 — Flow cytometry analysis of cell viability of permissive cells, under different experimental conditions. Description of data: (A) Flow cytometry-based analysis of propidium iodide uptake (FL-3) in pcDNA.3-, EGFP- and gelsolin-EGFP-transfected permissive cells (top panel, permissive lymphocyte cells; bottom panel, permissive HeLa cells) at 24 h post-transfection. Expression of EGFP- or gelsolin-EGFP is monitored in FL-1. Quantification of propidium iodide uptake (FL-3) by these treated cells (FL-1) is indicated in regions R of plots (represented as the percentage of total cells analyzed), per each experimental condition. A representative experiment of three is shown. (B) Flow cytometry-based analysis of propidium iodide uptake (FL-3) in scrambled and siRNA(1 + 2)-GSN-treated permissive cells (top panel, permissive lymphocyte cells; bottom panel, permissive HeLa cells) at 24 h post-nucleofection. Quantification of propidium iodide uptake (FL-3) by these treated cells (SSC, Side Scatter) is indicated in regions R of plots, per each experimental condition. A representative experiment of three is shown. In (A) and (B), not any significant toxicity is observed under each experimental condition. [file 1742-4690-10-39-S1.tiff]
